# Extending Validation of a Social Emotional Health Measure For Middle School Students

**DOI:** 10.1007/s40688-022-00411-x

**Published:** 2022-03-23

**Authors:** Michael J. Furlong, Jennica L. Paz, Delwin Carter, Erin Dowdy, Karen Nylund-Gibson

**Affiliations:** 1 University of California, Santa Barbara, CA USA; 2 Goleta, CA USA; 3 San Diego State University, San Diego, CA USA

**Keywords:** Social Emotional Healthy Survey, Covitality, Well-being, School mental health, Middle school

## Abstract

The Social Emotional Health Survey-Secondary-2020 (SEHS-S-2020) is a well-studied option for assessing social emotional health to support students within a multitiered system of school support. While a growing body of literature supports the SEHS-S-2020 measure for assessing student covitality, there is less validation evidence specifically for middle-school-aged students. The present study aimed to fill this gap in the literature by examining its use for younger adolescents. Study participants were from two samples, including a cross-sectional sample with 9,426 students in Grades 7–8 from 32 counties in California and a longitudinal sample with 414 students in Grades 6–8 from two middle schools. Data analyses examined structural validity, internal consistency, measurement invariance, criterion validity, predictive validity, and response stability. Results indicate excellent fit indices for a four-level higher-order measurement model, with adequate concurrent and one-year predictive validity coefficients, supporting the use of the SEHS-S-2020 measure with young adolescents in middle school settings. The discussion focuses on implications for assessing students’ psychosocial assets, universal school-based screening, and cultural and intersectionality considerations when interpreting SEHS-S-2020 responses.

**Supplementary Information:**

The online version contains supplementary material available at 10.1007/s40688-022-00411-x.

## Introduction

The worldwide effects of the Covid-19 pandemic have disrupted youths’ education and adversely impacted their mental health, with meta-analyses showing an increased prevalence of depression and anxiety disorders (Racine et al., 2021). Other surveillance reports emphasize the behavioral health challenges many youths are facing. Martinelli et al. (2020) reported overall decreasing youth well-being trends—72% of parents reported a decline in their child’s well-being. The biennial Youth Risk Behavior Surveillance Survey of US adolescents found a substantial increase of past year chronic sadness (41%) and serious suicidal ideation (36%) between 2009 and 2019 (Centers for Disease Control & Prevention, 2021). These experiences have raised concerns about youths’ social-emotional functioning (Murthy, 2021). Public policy and mental health experts emphasize the critical need to monitor mental well-being and provide equitable access to essential services to help build upon youths’ psychosocial strengths and mitigate traumatic experiences (e.g., American Academy of Pediatrics, 2021b; National Association of School Psychologists, 2021).

With the onset of nearly half of mental health diagnoses by age 14 (i.e., ADHD, anxiety, and depression; National Alliance on Mental Illness, 2021), middle school students are an age group particularly vulnerable to the adverse effects of the COVID-19 pandemic (National Association of Secondary School Principals, 2020). With the increased awareness of the need to monitor youths’ social and emotional health, especially among young adolescents in a vulnerable yet capable stage of life, it is crucial to recognize that schools are a natural ecosystem for these efforts to occur. Ideally, school-based mental health supports are provided in the context of a caring community that includes culturally affirming mental health professionals implementing a purposeful and comprehensive mental wellness program. The Social Emotional Health Survey-Secondary is a measure developed to support universal wellness screening. The SEHS-S has substantial psychometric research with high school students; however, it is not yet validated with middle-school-age adolescents. The present study fills this literature gap.

### Social Emotional Health Survey–Secondary (SEHS-S)

#### Description

Furlong and colleagues (2014) proposed that psychosocial strengths are related to a higher-order trait, covitality, contrasted with the mental health disorder comorbidity term. Covitality is, “the synergistic effect of positive mental health resulting from the interplay among multiple positive psychological building blocks” (Furlong et al., 2014, p. 3). The covitality principle considers psychosocial strengths as adaptive self-schemas linked with youth resilience and thriving developmental outcomes. These psychosocial strengths have the most impact when they co-occur in harmony rather than as isolation strengths (Furlong et al., 2020)*; the whole is greater than the sum of its parts.* Considered from a transactional development lens, fostering balanced development of multiple core psychosocial strengths (e.g., gratitude, empathy, and persistence) promotes positive interpersonal transactions within a child’s socio-ecological systems, contributing to optimal developmental outcomes (Furlong et al., 2020).

The covitality principle is operationalized with the 36-item Social Emotional Health Survey-Secondary (SEHS-S) measure that assesses 12 subscales assessing psychosocial strengths derived from the social emotional learning (SEL) and positive youth development (PYD) literature. The 12 subdomains are associated with four correlated positive social emotional health domains that assess the higher-order covitality latent construct. The first domain, belief-in-self, consists of three subscales grounded in constructs from self-determination theory literature: self-efficacy, self-awareness, and persistence. The second domain, belief-in-others, comprises three subscales derived from constructs found in childhood resilience literature: school support, peer support, and family support. The third domain, emotional competence, consists of three subscales based on constructs drawn from the SEL scholarship: emotion regulation, empathy, and behavioral self-control. The final domain, engaged living, comprises three subscales grounded in constructs derived from the positive youth psychology literature: gratitude, zest, and optimism. Research supports the cumulative resilience advantage as measured by the 12 SEHS-S subdomains. Students with more SEHS-S strengths report positive mental well-being and low levels of emotional risk behaviors (Lenzi et al., 2015a, 2015b, Moore et al., 2019). The SEHS-S research grounding and positive asset emphasis provide an alternative to emotional problem-focused universal school mental health screeners.

#### Previous Validation Studies

Since its development, 10 SEHS-S studies published in peer-reviewed journals have examined its reliability and validity (see Supplemental Material, Table 1 for SEHS validation studies). Three studies (Furlong et al., 2014; You et al., 2014, 2015) reported on its preliminary development with independent samples of California high school students. Confirmatory factor analyses supported a 1 → 4 → 12 measurement model with the 12 subscales treated as measured variables, loading on to four domains latent constructs (belief in self, belief in others, emotional competence, and engaged living) and one higher-order covitality latent construct. This model has been replicated with acceptable structural and concurrent validity model fit (SRMR, CFI, RMSEA) in six studies conducted in Japan (Iida et al., 2019; Ito et al., 2015), Korea (Lee et al., 2016), China (Pan et al., 2016), Lithuania (Ala et al., 2019), and Turkey (Telef & Furlong, 2017). Three of these studies included, but not exclusively, middle school-age students with older adolescents. In Spain, Piqueras et al. (2019) extended research by examining CFA fit statistics for the 1 → 4 → 12 → 36 model, which treated the 12 SEHS-S-2015 (see Furlong et al., 2018) subscales as latent constructs; analysis supported the hypothetical model’s structural validity, acceptable reliability, and concurrent validity. A study with an Iranian sample (Taheri et al., 2020) independently replicated the 1 → 4 → 12 → 36 model for the SEHS-S to be used as a self-report measure for older adolescents.

Recently, Furlong and colleagues (2020) developed an updated SEHS-S-2020 edition which standardized a four-point response scale (1 = *not at all true*, 2 = *a little true*, 3 = *pretty much true*, and 4 = *very much true*) for all 36 items and made minimal wording changes to enhance readability because the SEHS-S-2015 used a five-point response format for the zest and gratitude subscales (see Furlong et al., 2020). Drawing on the Piqueras et al. (2019) CFA analysis, two studies examined the structural validity of the 1 → 4 → 12 → 36 model for the revised SEHS-S-2020 version. Furlong et al. (2020) found an acceptable fit with high internal consistency, one-year stability, and concurrent validity with life satisfaction and emotional distress measures. However, middle-school-aged adolescents were not included in these previous analyses, highlighting the need for this current study.

### Study Purpose

The body of psychometric research validating the SEHS-S as a self-report measure for older adolescents is growing. However, there is less validation specifically for middle school adolescents (Grades 6–8, ages 11–13 in the USA), and there is limited validation evidence for the SEHS-S-2020 edition with this age group. Critically, there is a need to validate the SESH-S-2020 with middle school students because they are at a crucial development cusp with more diversity in physical and psychological development than in any other school context (Evans et al., 2018). Middle school campuses include pre/post-pubescent youths and those with varying higher-order association reasoning and emotional self-control (Qualter et al., 2007). The middle school years also present students with substantially increased demands for academic and social autonomy, and this age range is when many anxiety disorders emerge (Kessler et al., 2005). The physical, neuro-architectural, and behavioral changes that transpire during early adolescence create opportunities for students to become active agents in shaping their thriving developmental trajectories (National Academies and of Sciences, Engineering, and Medicine, 2019). Universal school wellness surveys provide youth with a vehicle to voice their social and emotional interests and needs. The developmental experiences of middle school students are sufficiently unique that we cannot assume that the SEHS-S-2020 structural model adequately captures their still-forming social and emotional competencies. The present study aimed to fill this gap in the SEHS-S-2020 validation literature to support its use across school configurations, including younger adolescents.

## Method

### Procedures and Participants

We examined the SEHS-S-2020’s psychometric characteristics drawing on subsets of data from the California Student Wellness Study (see www.covitalityucsb.info).

#### Sample 1, Cross-sectional Structural Validity

The California Healthy Kids Survey (CHKS) is an anonymous comprehensive school-based surveillance survey used in California for more than 20 years, administered by WestEd for the California Department of Education. A randomly selected subsample of students who completed the CHKS between October 2017 and June 2019 was used to examine structural validity. The CHKS survey responses used for the present study were funded by an Institute of Education Sciences grant and the data reported herein has not been used in any previous publication. Parents provided permission and students provided assent. A school-site administrator coordinates the CHKS online survey (see https://calschls.org/survey-administration/). Students complete the core CHKS module in Grades 7, 9, and 11. In some instances, schools opt to administer the survey to all students, allowing the evaluation of the SEHS-S-2020 with a middle-school-age school sample. The responses of middle school students in Grade 7 (84,057) and 8 (4,713) were compiled for the current study. The random sampling of 4,713 eighth graders equated the sample for analysis. The total sample size inclusive of seventh and eighth graders was 9,426 from 32 of California’s 58 counties across urban, suburban, and rural communities. In Sample 1, 50.5% identified as Latinx, and students indicated their gender identity as female (50.0%), male (48.6%), or declined to respond (1.4%). The characteristics of the data subsets used for calibration, validation, and invariance structural validity analyses are available in Supplemental Material, Table 2.

#### Sample 2, Concurrent and Predictive Validity, and Stability Analysis

Following university human subjects committee approval, passive parental consent, and student assent (electronically before the start of the survey administration), an online survey was administered at two California middle schools (ages 11–13 years) as part of their effort to monitor middle school students’ well-being. Teachers received a script with which to proctor administration. Students completed the online survey in October 2017 (Year 1) and October 2018 (Year 2). The survey presented the measures in the following order: SEHS-S-2020, SEDS, MSLSS, PANAS (see Measures section). The SEHS-S-2020 items were presented in a different random order to each student at each administration. We included Grade 6 students in the validity analyses because this information would interest educators in middle school configurations who use the SEHS-S-2020 with their entire student body. Each year, the students entered their unique school identifier, which allowed the examination of one-year response stability for 414 students. In Year 1, the students were in Grades 6 (31.4%), Grade 7 (31.6%), and Grade 8 (37.0%). The students’ preferred gender identification was female (51.9%), male (47.3%), and declined to state (0.7%). For ethnicity, most students identified as White (53.1%), two or more ethnicities (18.4%), Hispanic/Latinx (17.9%), and other ethnicities (10.3%). English (75.1%) was the home language for most students, followed by Spanish (15.0%) and another language (9.9%).

### Measures

Both samples completed the SEHS-S-2020. Sample 2 also completed the Brief Multidimensional Student Life Satisfaction Scale, Social Emotional Distress Scale, and the Positive and Negative Affect Scale for Children.

#### Samples 1 and 2: Social Emotional Health Survey-Secondary-2020

The SEHS-S-2020 includes 36 items as described earlier in this manuscript (items shown in Supplemental Material Table 4). The items use a four-point response format (1 = *not at all true*, 2 = *a little true*, 3 = *pretty much true*, 4 = *very much true*). The mean item responses across all 36 items for Sample 1 were as follows: Grade 7 (*M* = 3.03, *SD* = 0.59, skewness = -0.49, kurtosis = -0.15) and Grade 8 (*M* = 2.96, *SD* = 0.61, skewness = -0.45, kurtosis = -0.09). The mean differences by grade, *t* = 7.06 (11,424), *p* = 0.114, represented a negligible effect size difference (*d* = 0.13).

#### Sample 2: The Brief Multidimensional Student Life Satisfaction Sale (BMSLSS)

This widely used measure assesses student life satisfaction across friends, family, self, school, and living environment life domains. The response options are: 1 = *strongly dissatisfied* … 6 = *strongly satisfied* (Athay et al., 2012; Bickman et al., 2010). Acceptable internal consistency is reported for previous samples (α = 0.75–0.81; Huebner, 1991; Huebner et al., 2006). These are the reliability coefficients for Sample 2 (Year 1 α = 0.90; Year 2 α = 0.77).

#### Sample 2: Social Emotional Distress Scale (SEDS)

The 10 SEDS items assess adolescents’ recent (past month) emotional distress using a four-point response scale: 1 = *not at all true*, 2 = *a little true*, 3 = *pretty much true*, 4 = *very much true*. A sample item is, *I had a hard time breathing because I was anxious*. Previous CFA supports a unidimensional model with robust reliability α = 0.94 and ω = 0.95 (Dowdy et al., 2018). These are the reliability coefficients for Sample 2 (Year 1 and 2 α = 0.90).

#### Sample 2: Positive and Negative Affect Scale for Children (PANAS-C)

The PANAS-C (Ebesutani et al., 2012) assesses the frequency of past-week emotional experiences: 0 = *not at all*, 1 = *a little*, 2 = *moderately*, 3 = *quite a bit*, and 4 = *extremely*. The PANAS-C Positive Affect (PANAS–Pos; joyful, delighted, cheerful, alert, determined) and the PANAS-C Negative Affect (PANAS-Neg; scared, gloomy, nervous, upset, sad) have demonstrated adequate reported alpha reliability coefficients of 0.76 and 0.85, respectively. These are Sample 2’s reliability coefficients for Positive Affect (Year 1 α = 0.72, Year 2 α = 0.73) and Negative Affect (Year 1 α = 0.84, Year 2 α = 0.83).

### Data Analysis Plan

#### Sample 1: Cross-sectional Structural Validity Analysis Plan

*Conformatory Factor Analysis (CFA).* CFA with the SEHS-S-2020 evaluated support for its hypothesized 1 → 4 → 12 → 36 higher-order model. Model fit was assessed using recommendations from the literature: comparative fit index (CFI > 0.95), root mean square of approximation (RMSEA < 0.05), and standardized root mean square residual (SRMR < 0.05) indicated excellent model fit (Browne & Cudeck, 1989; Hu & Bentler, 1999). Using Mplus 8 version 8.4 (Muthén & Muthén, 1998–2019) cross-validation (CV) was conducted on a random subsample of 2000 students (1000 seventh graders and 1000 eighth graders) drawn from the 9,426 Sample 1 students to evaluate the full 1 → 4 → 12 → 36 covitality model (Whittaker & Stapleton, 2006). The use of cross-validation is important when selecting a reliable model expected to fit data from other samples (MacCallum et al., 1992; Whittaker & Stapleton, 2006). The full 1 → 4 → 12 → 36 covitality CFA model was estimated with a random subsample of *n* = 1,000 (i.e., subsample 1-A). A second random subsample of *n* = 1,000 cases (500 seventh graders and 500 eighth graders; subsample 1-B), drawn without replacement, was estimated to replicate model fit. Next, parameters from the subsample 1-A full covitality CFA model were used as fixed values to estimate the model with subsample 1-B. Information criteria (i.e., AIC, BIC, SABIC) were retained from both the freely estimated model and the model with fixed values (i.e., subsample 1-B_free_ and subsample 1-B_fixed_). Findings that the model with fixed values produces the lower information criteria values supports the validity of the full 1 → 4 → 12 → 36 factorial model. Lastly, the process was repeated with subsample 1-A fixed to the parameters from subsample 1-B being compared to subsample 1-A freely estimated (i.e., subsample 1-A_fixed_ and subsample 1-A_free_). Finding lower information criteria values in subsample 1-A_fixed_ provides further evidence of robust model replicability.

##### Internal Consistency Analysis Plan

SEHS-S-2020 Cronbach’s alpha (α) and omega (ω) coefficients were evaluated for its 12 subdomains, 4 domains, and the overall covitality index. Values higher than 0.80 provide evidence that the items are measuring the same construct (Cronbach, 1951, McDonald, 1999).

##### Measurement Invariance (MI) Analysis Plan

To evaluate SEHS-S-2020 score invariance across a range of demographic subgroups, multigroup CFA examined MI for (a) gender, (b) grade level, (c) Hispanic/Latinx status, and (d) ethnicity identification. This analysis used Mplus version 8.4 (Muthén & Muthén, 1998–2021) with maximum likelihood (ML) and unit variance identification. Using random subsamples of *n* = 1000 from the structural validity Sample 1, CFAs analyzed model fit for subgroups. Subsequently, successive multigroup CFAs were employed to evaluate configural, metric, and scalar invariance (Vandenberg & Lance, 2000). MI provides evidence that the factor structure, loadings, and intercepts are similar across subgroups. Invariance tests, conducted sequentially, first examined the model with all parameters freely estimated across groups (configural invariance). Determining configural invariance establishes that the model’s structure fits the data well for each compared group. Next, metric invariance was tested by holding the loadings equal across groups. When compared to the configured models, metric invariance is established when ΔCFI < 0.01 and ΔRMSEA < 0.015 (or ΔSRMR < 0.03; Chen, 2007). Scalar invariance analysis held the loadings and intercepts equal across groups. The establishment of scalar invariance indicates that participants’ scores on the latent construct and observed variable will be the same regardless of their group membership. Scalar invariance is confirmed when the comparison to the metric model yields a ΔCFI < 0.01 and ΔRMSEA < 0.015 (or ΔSRMR < 0.03) (Chen, 2007). Scalar invariance, when found, allows researchers to make inferences via extrapolation claims for each of the subgroups.

#### Sample 2: Concurrent and Predictive Validity, and Stability Analysis Plan

An ANOVA compared the mean SEHS-S-2020 total covitality scores across the Grade 6, 7, and 8. Bivariate validation Pearson correlations examined association of the total covitality score with concurrent and one-year predictive measures. These analyses were computed with SPSS v28.01.

## Results

### Sample 1: Cross-sectional Structural Validity Results

#### Conformatory Factor Analysis

The CFA for the SEHS-S-2020 1 → 4 → 12 → 36 hypothesized higher-order factor structure had excellent model fit, χ2(578) = 11,156.85, *p* < 0.001, CFI = 0.956, RMSEA = 0.043 [CI = 0.042, 0.043], and SRMR = 0.045. The calibration and validation results with subsamples 1-A and 1-B indicated an almost identical model fit and lower information criteria values, providing evidence that the full covitality model was successfully replicated with a different subsample (see Table 1).

**Table 1:** Sample 1 Double Cross-Validation of the Full SEHS-S-2020 Hypothesized Model in Middle School Students

Model	AIC	BIC	SABIC	*LL*	*LL*_*diff*_	nPAR	Δ*df*	*p*
Subsample 1-A_free_	73,575.43	74,183.99	73,790.16	-36,663.72		124		
Subsample 1-A_fixed_	73,638.88	**73,638.88**	**73,638.88**	-36,819.44	311.45	0	124	< .001
Subsample 1-B_free_	75,448.91	76,057.47	75,663.64	-37,699.45		124		
Subsample 1-B_fixed_	75,546.78	**75,546.78**	**75,546.78**	-37,773.39	345.88	0	124	< .001

AIC = Akaike Information Criterion. BIC = Bayes Information Criterion. SABIC = Sample Size Adjusted Bayes Information Criterion. nPAR = Number of Free Parameters

#### Internal Consistency Analysis

SEHS-S-2020 Cronbach’s alpha (α) and Omega (ω) coefficients were evaluated for its 12 subdomains, 4 domains, and the overall covitality index for Sample 1. The SEHS-S-2020 covitality total score internal consistency was excellent (α = 0.96, ω = 0.95). The four SEHS-S-2020 domains showed excellent reliability (BIS α = 0.88, ω = 0.87; BIO α = 0.87, ω = 0.85; EC α = 0.87, ω = 0.87; EL α = 0.94, ω = 0.93), and subscale coefficients indicated moderate to strong reliability (α range = 0.70–0.95, ω range = 0.70–0.95, see Supplemental Material, Table 3 for all reliability coefficients).

#### Measurement Invariance (MI) Analysis

Initial CFAs for each group and subgroup indicated an excellent fit. Tests for MI indicated that all three levels of the model were invariant across: (a) grade level (i.e., Grades 7 and 8, see Table 2); (a) gender (i.e., male v. female binary identity, see Table 3); and (c) Hispanic/Latinx identification (i.e., Hispanic/Latinx or non-Hispanic/Latinx, see Table 4). The ΔCFI was less than 0.01, ΔRMSEA < 0.015, and ΔSRMR < 0.03 for all comparisons for all groups. Results indicated that the SEHS-S-2020 items measure the covitality construct in similar ways across relevant demographic identifications, supporting future extrapolation and scoring claims.

**Table 2:** Sample 1 Invariance Across Grade

Model	χ2	*df*	Δχ2	Δ*df*	RMSEA	90% RMSEA CI	CFI	SRMR	ΔCFI	ΔRMSEA	ΔSRMR
*CFA*											
Both	10,038.69	578	—	—	.042	[.041, .042]	.959	.042	—	—	—
7^th^ Grade	5272.16	578	—	—	.042	[.040, .043]	.957	.043	—	—	—
8^th^ Grade	5539.35	578	—	—	.043	[.042, .044]	.958	.043	—	—	—
*MI Level 1*											
Configural	7946.91	1056	—	—	.037	[.036, .038]	.970	.032	—	—	—
Metric	8017.91	1092	71.00	36	.037	[.036, .037]	.970	.037	< .001	< .001	.005
Scalar	8270.81	1128	252.90	36	.037	[.036, .037]	.969	.041	.001	< .001	.003
*MI Level 2*											
Configural	10,688.72	1176			.041	[.041, .042]	.958	.042	—	—	—
Metric	10,784.16	1212	95.44	36	.043	[.041, .042]	.958	.043	< .001	.002	.001
Scalar	10,914.12	1224	129.96	12	.041	[.040, .042]	.958	.044	< .001	.002	.001
*MI Level 3*											
Configural	10,994.81	1188	—	—	.042	[.041 .043]	.957	.043	—	—	—
Metric	11,091.77	1224	96.96	36	.041	[.041, .042]	.957	.043	< .001	.001	< .001
Scalar	11,172.12	1228	80.35	4	.041	[.041, .042]	.957	.045	< .001	< .001	.002

CFA = Confirmatory Factor Analysis. Level 1 refers to invariance for lower-order factors. Level 2 refers to the second-order factors, and Level 3 refers to the higher-order factor

**Table 3:** Sample 1 Invariance Across Gender

Model	χ2	*df*	Δχ2	Δ*df*	RMSEA	90% RMSEA CI	CFI	SRMR	ΔCFI	ΔRMSEA	ΔSRMR
*CFA*											
Both	10,038.69	578	—	—	.042	[.041, .042]	.959	.042	—	—	—
Male	5011.42	578	—	—	.041	[.040, .042]	.960	.043	—	—	—
Female	5404.17	578	—	—	.042	[.041, .043]	.958	.041	—	—	—
*MI Level 1*											
Configural	7794.03	1056	—	—	.037	[.036, .038]	.970	.032	—	—	—
Metric	7986.74	1092	192.71	36	.037	[.036, .038]	.969	.038	.001	< .001	.006
Scalar	9582.10	1128	1595.36	36	.040	[.039, .041]	.962	.050	.007	.003	.012
*MI Level 2*											
Configural	10,469.32	1176	—	—	.041	[.041, .042]	.959	.041	—	—	—
Metric	10,619.76	1212	150.44	36	.041	[.040, .042]	.958	.042	.001	< .001	.001
Scalar	12,141.98	1224	1522.22	12	.044	[.043, .045]	.952	.047	.006	.003	.005
*MI Level 3*											
Configural	11,826.70	1188	—	—	.044	[.043, .045]	.953	.045	—	—	—
Metric	11,970.08	1224	143.38	36	.043	[.043, .044]	.952	.046	.001	.001	.001
Scalar	12,404.39	1228	434.31	4	.044	[.044, .045]	.950	.048	.002	.001	.002

CFA = Confirmatory Factor Analysis. Level 1 refers to invariance for lower-order factors. Level 2 refers to the second-order factors, and Level 3 refers to the higher-order factor

**Table 4:** Sample 1 Invariance Across Hispanic/Latinx Identification

Model	χ2	*df*	Δχ2	Δ*df*	RMSEA	90% RMSEA CI	CFI	SRMR	ΔCFI	ΔRMSEA	ΔSRMR
*CFA*											
Both	10,038.69	578	—	—	.042	[.041, .042]	.959	.042	—	—	—
Hispanic	5346.77	578	—	—	.042	[.041, .043]	.958	.042	—	—	—
NonHispanic	5453.40	578	—	—	.043	[.042, .044]	.957	.043	—	—	—
*MI Level 1*											
Configural	7932.40	1056	—	—	.037	[.037, .038]	.970	.031	—	—	—
Metric	8001.15	1092	68.75	36	.037	[.036, .038]	.969	.036	.001	< .001	.005
Scalar	8485.20	1128	484.05	36	.037	[.037, .038]	.967	.041	.002	< .001	.005
*MI Level 2*											
Configural	10,850.41	1176	—	—	.042	[.041, .043]	.957	.042	—	—	—
Metric	10,934.91	1212	84.50	36	.042	[.041, .042]	.957	.043	< .001	< .001	.001
Scalar	11,128.33	1224	193.42	12	.042	[.041, .042]	.956	.045	.001	< .001	.002
*MI Level 3*											
Configural	11,113.93	1188	—	—	.042	[.042, .043]	.956	.043	—	—	—
Metric	11,195.27	1224	81.34	36	.042	[.041, .043]	.956	.043	.001	< .001	< .001
Scalar	11,370.66	1228	175.39	4	.042	[.041, .043]	.955	.046	.001	< .001	.003

CFA = Confirmatory Factor Analysis. Level 1 refers to invariance for lower-order factors. Level 2 refers to the second-order factors, and Level 3 refers to the higher-order factor

### Sample 2: Concurrent and Predictive Validity, and Stability Results

#### Reliability and Stability

An ANOVA, using SPSS V28.01, compared the Sample 2 mean SEHS-S-2020 total covitality scores across Grades 6, 7, and 8. The Year 1 mean item responses for all grades were negatively skewed (Grade 6 M = 3.23, *SD* = 0.48, Grade 7 M = 3.21, SD = 0.43; Grade 8 M = 3.09, *SD* = 0.44) and significantly different, *F*(2.411) = 4.46, *p* = 0.012; Levine (2, 411) = 0.53, *p* = 0.587, but the effect size of the difference was small (η 2 = 0.012). The Year 2 mean item responses for all grades were also negatively skewed (Grade 6 M = 3.16, *SD* = 0.47, Grade 7 M = 3.08, *SD* = 0.46; Grade 8 M = 3.09, *SD* = 0.44) but not significantly, *F* (2.411) = 1.24, *p* = 0.291; Levine (2, 411) = 0.091, *p* = 0.913. The internal consistency of the covitality total score for Years 1 and 2 was α = 0.94, with one-year test–retest coefficient of *r* = 0.66.

#### Concurrent and Predictive Validity

Bivariate correlations examined the association of the total covitality score with concurrent and one-year predictive measures for Sample 2. All concurrent BMSLSS (*r* = 0.65), PANAS-Pos (*r* = 0.59), SEDS (*r* = -0.46), PANAS-Neg (*r* = -0.38) and one-year predictive validity coefficients BMSLSS (*r* = 0.52), PANAS-Pos (*r* = 0.43), SEDS (*r* = -0.27), PANAS-Neg (*r* = -0.27) were significant (*p* < 0.001) in the expected directions. The concurrent validity coefficients had adequate (PANAS-Neg) or considerable correspondence (BMSLSS, PANAS-Pos, SEDS) with adequate one-year prediction for the BMSLSS and PANAS-Pos wellness indicators (Shepherd et al., 2015).

## Discussion

Given the increased awareness of the need to focus on student social and emotional health, it is critical to have a measure that supports efforts to assess mental health and wellness in schools. Considering the developmental changes experienced by middle-school-age students and the continued emphasis on early identification and prevention, practitioners and researchers now have a tool to guide mental wellness efforts in younger adolescents. Consistent with previous SEHS-S-2020 research among older adolescents, this study provides psychometric evidence supporting its use with early adolescents.

This study supported the higher-order covitality model (1 → 4 → 12 → 36); hence, the overall covitality index, the four domains, and the 12 subscales can be used with middle-school-age students. This important finding indicates that it is possible to analyze, for each student or a schoolwide prevention effort, the 12 psychological building blocks and four higher-order domains that have robust evidence of being linked to positive youth development. In alignment with best-practice intervention principles, preventative techniques may be helpful to boost any of the 12 latent traits (Lenzi et al., 2015a, 2015b). For example, following the administration of the SEHS-S-2020, a practitioner should feel confident in their ability to assess a student’s level of peer support, emotional regulation, gratitude, and each of the 12 latent traits.

The invariance findings indicate that the covitality construct is measured similarly across important gender, grade, and ethnic identification. This finding is essential for schoolwide efforts when assessing students from diverse backgrounds together. Additionally, stability findings for this sample of middle school students are like findings of older adolescents (Furlong et al., 2020) and suggest that psychological strengths form early and are generally stable. These findings underscore the need for early efforts to help students develop positive internal and external working models. The importance of monitoring covitality and constructs assessed on the SEHS-S-2020 is also essential considering their relations to significant life outcomes, including increased academic performance, life satisfaction and positive affect, and decreased negative affect and social-emotional distress.

### SEHS-S-2020 Interpretation Considerations

The present findings contribute to the body of validation research supporting the use of the SEHS-S-2020 to assess meaningful adolescent psychological mindsets for various groups of students (e.g., age, gender identity, and sociocultural identity). Its psychometric properties are robust and support individual clinical and wellness surveillance. The SEHS-S-2020 was added to the California Healthy Kids core module's during the 2021–2022 academic year, further emphasizing the importance of this measure being examined for use with middle school age students. Additionally, schools in eight US states and in Japan (Iida et al., 2019), Chile (Varela et al., 2022), Spain (Piqueras et al., 2019) use it for universal screening to inform school-based mental health research and services. As the SEHS-S-2020 use expands, we offer three considerations for thoughtful interpretation.

### Consideration 1: What are the Student’s Other Developmental Assets?

The SEHS-S-2020 36 items assess latent constructs linked to core developmental task domains. These constructs draw upon theoretical perspectives from social psychology, self-determination theory, developmental assets literature, social-emotional learning literature, and positive psychology literature. The four domains pertain to the life-long coalescence of a person’s sense of self, social belonging, emotional management, and positive character traits. When using the SEHS-S-2020, it is crucial to recognize that its 12 subscales and four domains do not include all conceivable student developmental assets. Thoughtful mental wellness evaluations will also need to consider other student positive dispositions, competencies, and cultural assets.

Other constructs could provide meaningful options in specific sociocultural contexts. For example, gratitude is in the engaged living component of the SEHS-S-2020. In some cultural contexts, such as Korea and China (Mendonça et al., 2018), gratitude has nuanced interpretations. Emmons et al.’s (2019) definition of gratitude includes a moral component that presumably increases a Western culture person’s motivation to reciprocate but falls short of a repaid social debt. The three blessings gratitude exercise shows that gratitude is boosted merely by reflecting daily on benefits received from others without planning or engaging in reciprocal actions. However, in some cultural contexts, reflecting on benefits received from others without reciprocity could produce guilt feelings for accepting benefits and not giving in return.

Another consideration is that various social, emotional, and social-cultural experiences can be associated with other meaningful constructs that fit the covitality principle. In China, the concept of psychological suzhi (Qian et al., 2020) has similarities with the covitality principle but has a deeper meaning within Chinese cultures. In a similar vein, Hispanic/Latinx cultures might prioritize evaluating different assets such as academic persistence and *familismo* (Hernandez et al., 2021). In Hawai’i, the statewide SEL framework has an important focus on the indigenous Hawaiian values, language, culture, and history, and students’ sense of belonging and responsibility to the *Āina* (land) of Hawai’i) as a valued “place” (Hawai’i State Department of Education, n.d., 2019). Predating Seligman’s positive psychology initiative (Seligman & Csikszentmihalyi, 2000), Black American psychologists, such as Joe White (1984), identified seven psychological strengths uniquely shaped by African Americans’ experiences of slavery and racism: improvisation, resilience, connectedness to others, spirituality, emotional vitality, gallows of humor, and a “healthy suspicion of you know who” (White, 1984). White described emotional vitality as having excitement, a high level of energy, zest for life—behaving in a manner that approaches life enthusiastically. White’s conceptualization aligns closely with the SEHS-S-2020 zest subscale, yet we caution that its three items do not adequately capture the nuanced expression that White intended. Finally, with only three items per subscale, the SEHS-S-2020 subscales are inadequate when researcher and intervention interests are narrowly focused, for instance, on student optimism, persistence, and self-regulation.

### Consideration 2: Are the SEHS-S-2020 Assets Having a Protective Effect?

As a growing body of research identifies various development benefits associated with high covitality (Lenzi et al., 2015a, 2015b), we caution that more research is needed to examine various developmental outcomes among vulnerable student groups. The documented coping and protective effects of the SEHS-S-2020 might not manifest equally for all students. As an example, sexual-minoritized youth with intersectional identities report substantially higher suicide ideation than their cisgender peers, particularly when they experience gender-related, bias, and victimization (Turban et al., 2021). Conversely, other research indicates transgender youth experience positive mental health when their gender identity is supported and validated within their social spheres (Olson et al., 2016), highlighting social support as a protective factor. Examining this dynamic, O’Malley et al. (2021) evaluated the SEHS-S-2020 constructs’ resilience-enhancing potential for students who experienced bullying and victimization due to gender identity and sexual orientation. For students identifying as transgender and experiencing minority-related bullying and victimization, O’Malley et al. (2021) found that covitality strengths, measured by the SEHS-S-2020, did not protect against suicide ideation or chronic sadness. That is, evidence for the covitality principle effect was not found. Although the O’Malley et al. and other studies have found promotive and protective developmental covitality effects, it does not follow that this is necessarily the case for all students, particularly those who have life experiences subject to historical othering and oppression. For some students, the intensity of exposure to trauma and identity-related victimization could be so pervasive and upsetting that, as measured by the SEHS-S-2020, covitality’s resilience advantage is overwhelmed. The importance of this consideration is that well-intentioned school-based services focusing on efforts to foster individual student assets, metaphorically activating the covitality principle, may fall short. Instead, we advocate for a balanced perspective that recognizes a parallel effort to create safe and affirming social environments for all students to reduce exposure to warfare, trauma, racism, harassment, and other forms of oppression that could overwhelm their assets’ resilience capacity (Edwards, 2021).

### Consideration 3: How do Students Use their Strenghts?

The SEHS-S-2020 covitality elements are associated with overall personal development, with several studies showing positive associations with global subjective well-being. High subjective well-being is only one outcome indicator; future research must explore the broader purpose of fostering covitality strengths. The covitality principle, as measured by the SEHS-S-2020, signifies that a student has a comprehensive set of social and psychological assets; however, it does not provide information about how the student uses those assets. The links between the covitality principle and youths’ broader purposes, dreams, and aspirations are yet unexamined sufficiently. What values do they hold? How do they see themselves as global citizens? How do they contribute meaningfully to their micro- and macro-communities (Mercier et al., 2019) via the arts, activism, public service, or community building? Thoughtful social emotional assessment will include a validated measure like the SEHS-S-2020, and measures that seek to understand what the student values and the life trajectories.

### Study Qualifications

A primary qualification of this study’s findings is that although the sample size was large and drawn from urban, suburban, and rural communities, it is not representative of the wider USA middle school student population. In the California public education context, students who identify as Latinx comprise a plurality of students, as noted in the Participants description section of the manuscript. Hence, establishing measurement invariance for Latinx-identifying students is a prerequisite for use in the California school context, a substantial world geographic and population center. As apparent in the history of slavery and racism in the USA, questions about race and ethnic-cultural identification are fraught with social over-tones. California has one of the most diverse populations worldwide, having drawn citizens from Europe, Central America, and the Pacific Rim for more than 150 years. Hence, the statewide school survey providing the current study’s data set asks three questions about race. The “race” question uses categories employed by the US national census: American Indian/Alaskan Native, Asian, Black, White, Native Hawaiian/ Pacific Islander. Students can select one or more “racial” groups. Almost one-half (47.2% in the invariance sample) of the students reported identifying with two or more “racial” groups, attesting to the unique diversity of California’s student population.

Given this sample demographic characteristic and size, the proportion of students in other important groups, such as those identifying a Black, was small. Furthermore, the use of historical “racial” terms is reductionist. For example, the California statewide survey asks students who identify as racially “Asian” for more specific information with the following groups represented: Asian Indian, Cambodian, Chinese, Filipino, Hmong, Japanese, Korean, Laotian, Vietnamese, and other Asian. Hence, efforts to examine appropriate use for all these groups would require a large dataset and are much needed.

We further note the complexity of using “race” to indicate students’ relevant social contexts when intersectionality is prominent in diverse social contexts like California. Notably, in other California statewide surveys, 65% of students who identified with two or more “racial” groups also identified as Hispanic, yet 25% of students who identified as White also identified as Hispanic. Even 11% of American Indian students identified as Hispanic. Intersectionality matters related to “race” and ethnicity are the norm in the highly diverse California social context. We acknowledge these limitations and recognize the need for future research to evaluate the SEHS-S structural validity, especially among historically underrepresented or minoritized groups.

Since this study employed mono-method procedures (i.e., self-report), future studies should investigate multi-informant assessments (parent and teacher forms) based on the covitality framework. Another limitation is that some types of validation were unexamined. It is essential to evaluate how students’ responses to the SEHS-S-2020 are associated with their daily school experiences. Research examining other measurement methods (e.g., experience sample monitoring) is needed to investigate the association between students’ real-time emotions with characteristics measured by the SEHS-S-2020.

## Conclusion

The SEHS-S-2020 is available for use as part of comprehensive school-wide efforts to respond to the need for students’ mental and behavioral health services. Structural validity, internal consistency, measurement invariance, criterion validity, predictive validity, and response stability estimates all support its use among diverse young adolescents in middle schools. The SEHS-S-2020 can be helpful to assess students’ psychosocial assets as part of school-based screening efforts to support students’ well-being.

## Supplementary Information

Below is the link to the electronic supplementary material.

Supplementary file1 (DOCX 45 kb)
