# Supplementary material for: Extending Validation of a Social Emotional Health Measure For Middle School Students
Source: Contemp Sch Psychol. 2022 Mar 23;27(1):92–103. doi: 10.1007/s40688-022-00411-x (PMC8941839; doi:10.1007/s40688-022-00411-x)
Supplement: Supplementary file 1 — Supplementary file1 (DOCX 45 kb) [file 40688_2022_411_MOESM1_ESM.docx]

**Online Supplemental Material**

**Supplemental Material Table 1: Social Emotional Health Survey–Secondary (SEHS-S) Psychometric Validation Studies**

| Study | Sample | Grade/Age | Model | SRMR | CFI | RMSEA | Invariance | Covitality Reliability | Validity |
| --- | --- | --- | --- | --- | --- | --- | --- | --- | --- |
| SEHS-S 2015 | |  |  |  |  |  |  |  |  |
| Furlong et al. (2014) | California *N*=4,189 72% Hispanic/Latinx  51% female | Gr. 8, 10, 12, 12 schools | 1=>4=>12 | .05 | .92 | .07 | Binary gender | NA | SSLSS, PANAS |
| You et al. (2014) | California *N*=2,440 72% Hispanic/Latinx  53% female | Gr. 9-12 2 schools | 1=>4=>12 | .05 | — | .06 | Binary gender by ages 13-15 vs. 16-18 | NA | BESS, GPA |
| You et al. (2015) | California *N*=14,171 58% Hispanic/Latinx, 51% female | Gr. 9-12 17 schools | 1=>4=>12 | .04 | — | .08 | White, Black, Asian,  Hispanic/Latinx, Blended identity | α at least .95 for all groups | NA |
| Ito et al. (2015) | Japan *N*=975 | Gr. 7-9 2 schools | 1=>4=>12 | .05 | — | .07 | Binary gender | NA | SSLSS, PANAS |
| Lee et al. (2016) | Korea *N*=716 56% female | Age 13-18,  13 schools | 1=>4=>12 | .05 | .98 | .08 | Binary gender | NA | SSLSS |
| Pan Yan-Gu et al. (2016) | China *N*=726 55% female | 3 middle,  1 high school | 1=>4=>12 | .05 | .92 | .08 | NA | NA | NA |
| Telef et al. (2017) | Turkey *N*=827 55% female | Gr. 9-12 8 schools | 1=>4=>12 | .04 | .96 | .04 | NA | α M=.89 α F=.81 | SDQ, RSCA |
| Ala et al. (2019) | Lithuania *N*=935 52% male | Age 12-15, 16-18 | 1=>4=>12 | .05 | .94 | .08 | NA | α=.80 | IRI, RSE |
| Iida et al.  (2019) | Japan *N*=3,044 Test-retest *N*=106  55% female | 4 schools | 1=>4=>12 | NA | .95 | .04 | Binary gender | 3-wk test-retest =.79 | SCC, BMSLSS, SEDS |
| Piqueras et al. (2019) | Spain *N*=1,042 58% male | Gr. 7-10,  11-12, 8 schools | 1=>4=>12 =>36 | .05 | .96 | .03 | Binary gender | ω=.87 α=.93 | SDQ, MHC-SF, KIDSCREEN |
| SEHS-S-2020 | |  |  |  |  |  |  |  |  |
| Furlong et al. (2020) | California *N*=72,740 51% female Validity *N*=10,757  Test-retest *N*=707 | Gr. 9-12  113 schools | 1=>4=>12 =>36 | .05 | .96 | .04 | Binary gender,  5 sociocultural groups, full invariance | ω=.95  1-year test-retest =.68 | BMSLSS, SEDS |
| Hinton et al. (2020) | California *N*=1,404 55% female | Gr. 9-12 | 1=>4=>12 =>36 | .05 | .95 | .05 | Spanish vs.  English forms, full invariance | NA | NA |
| Taheri et al. (2020) | Iran *N*=373 100% female | Gr. 11-12 1 school | 1=>4=>12 =>36 | .05 | .94 | .04 | NA | α .81 | ESS, EPOCH |

*Note.* BESS = Behavioral and Emotional (Kamphaus & Reynolds, 2011). BMSLSS = Brief Multidimensional Life Satisfaction Scale (Huebner et al., 2006). Covitality = SEHS-S Covitality total score. EPOCH = EPOCH Measure of Adolescent Well-Being Kern et al., 2016). BESS = Examination Stress Scale. GPA = Grade Point Average. IRI = Interpersonal Reactivity Index (Hawk et al., 1997). KIDSCREEN (2006). MHC-SF = Mental Health Continuum-Short Form (Keyes, 2006). PANAS = Positive and Negative Affect Scale (Ebesutani et al., 2012). RSE = Rosenberg Self-Esteem Scale (1989). RSCA = Resilience Scale Children & Adolescents (Keyes, 2006). SCC = School Connectedness Scale (Furlong et al., 2011). SEDS = Social Emotional Distress Scale (Dowdy et al., 2018). SDQ = Strengths and Difficulty Questionnaire (Goodman, 1997). SSLSS = Student School Life Satisfaction (Huebner, 1991). RSCA (Prince-Embury, 2008). In the 1=> 4=> 12 CFA model the 12 SEHS-S component subscales (e.g., self-efficacy, gratitude) are treated as measures variable. In the 1=> 4=> 12=> 36 CFA model, the 12 SEHS-S component subscales (e.g., self-efficacy, gratitude) are treated as latent variables, each measured with three items.

Dowdy, E., Furlong, M. J., Nylund-Gibson, K., Moore, S., & Moffa, K. (2018). Initial validation of the Social Emotional Distress Scale to support complete mental health screening. *Assessment for Effective Intervention, 43*, 241–248. <https://doi.org/10.1177%2F1534508417749871>

Ebesutani, C., Regan, J., Smith, A., Reise, S., Higa-McMillan, C., Chorpita, B. F. (2012). The 10-item positive and negative affect schedule for children, child and parent shortened versions: Application of item response theory for more efficient assessment. Journal of *Psychopathology and Behavioral Assessment, 34*(2), 191–203. <https://doi.org/10.1007/s10862-011-9273-2>

Furlong, M. J., O’Brennan, L. M., & You, S. (2011). Psychometric properties of the Add Health School Connectedness Scale for 18 sociocultural groups. *Psychology in the Schools, 48,* 986–997. <http://dx.doi.org/10.1002/pits.20609>

Goodman, R. (1997). The Strengths and Difficulties Questionnaire: A research note. *Journal of Child Psychology and Psychiatry, 38,* 581–586. <https://doi.org/10.1111/j.1469-7610.1997.tb01545.x>

Hawk, S. T., Keijsers, L., Branje, S. J., Graaff, J. V. D., Wied, M. D., & Meeus, W. (2013). Examining the interpersonal reactivity index (IRI) among early and late adolescents and their mothers. *Journal of Personality Assessment, 95*(1), 96–106. <https://doi.org/10.1080/00223891.2012.696080>

Huebner, E. S. (1991). Further validation of the students’ life satisfaction scale: The independence of satisfaction and affect ratings. *Journal of Psychoeducational Assessment, 9*, 363–368. <https://doi.org/10.1177%2F073428299100900408>

Huebner, E. S., Seligson, J. L., Valois, R. F., & Suldo, S. M. (2006). A review of the brief Multidimensional Students’ Life Satisfaction Scale*. Social Indicators Research, 79*(3), 477–484. <https://www.jstor.org/stable/27522650>

Kamphaus, R. W., & Reynolds, C. R. (2012). *Behavior Assessment System for Children—Third Edition (BASC-2): Behavioral and Emotional Screening System (BESS)*. Bloomington, MN: Pearson.

Kern, M. L., Benson, L., Steinberg, E. A., Steinberg, L. (2016). The EPOCH measure of adolescent well-being. *Psychological Assessment, 28,* 586–597. <https://psycnet.apa.org/doi/10.1037/pas0000201>

Keyes, C. L. M. Mental health in adolescence: Is America’s youth flourishing? *American Journal* *of Orthopsychiatry, 76,* 395–402. <https://doi.apa.org/doi/10.1037/0002-9432.76.3.395> *8,* 586–597. <https://psycnet.apa.org/doi/10.1037/pas0000201>

KIDSCREEN Group Europe. (2006). *The KIDSCREEN questionnaires. Quality of life questionnaires for children and adolescents.* Pabst Science.

Prince-Embury, S. (2008). The resiliency scales for children and adolescents, psychological symptoms, and clinical status in adolescents. *Canadian Journal of School Psychology, 23*(1), 41–56. <https://doi.org/10.1177%2F0829573508316592>

Rosenberg, M. (1989). *Society and the adolescent self-image. Revised edition*. Wesleyan University Press.

**Supplemental Material Table 2. Cross-sectional Sample Descriptive Information**

| Sample Descriptive Information | Calibration Subsample 1-A  *n* = 1,000 | | Validation Subsample 1-B  *n* = 1,000 | | | Invariance Sample 1  *N* = 9,426 | |
| --- | --- | --- | --- | --- | --- | --- | --- |
|  | *n* | % | *n* | % | | *n* | % |
| *Grade* |  | |  | | |  | |
| Grade 7 | 500 | 50.0 | 500 | 50.0 | | 4713 | 50.0 |
| Grade 8 | 500 | 50.0 | 500 | 50.0 | | 4713 | 50.0 |
| *Gender identification* |  |  |  |  | |  |  |
| Male | 488 | 48.8 | 482 | 48.2 | | 4585 | 54.2 |
| Female | 502 | 50.2 | 506 | 50.6 | | 4702 | 44.0 |
| No response | 10 | 1.0 | 12 | 1.2 | | 139 | 1.5 |
| *Ethnic identification* |  |  |  |  | |  |  |
| American Indian, Alaskan Native | 33 | 3.3 | 32 | 3.1 | | 299 | 3.2 |
| Asian | 130 | 13.0 | 127 | 12.7 | | 1193 | 12.7 |
| Black, African American | 25 | 2.5 | 29 | 2.5 | | 289 | 3.1 |
| Native Hawaiian, Pacific Islander | 7 | 0.7 | 7 | 0.7 | | 78 | 0.8 |
| White | 257 | 25.7 | 262 | 25.7 | | 2463 | 26.1 |
| Multiple ethnic identity (2 or more) | 464 | 46.4 | 481 | 48.1 | | 4446 | 47.2 |
| Missing | 84 | 8.4 | 62 | 6.2 | | 658 | 7.0 |
| *Hispanic identification* | |  |  |  | |  |  |
| Non-Hispanic/Latinx | 480 | 48.0 | 492 | 49.2 | | 4610 | 48.9 |
| Hispanic/Latinx | 511 | 51.1 | 497 | 49.7 | | 4691 | 49.8 |
| Missing | 9 | 0.9 | 11 | 1.1 | | 125 | 1.3 |
| *Parent education (highest parent)* | | | | |  |  |  |
| Did not finish high school | 84 | 8.4 | 78 | 7.8 | | 798 | 8.5 |
| Graduated high school | 138 | 13.8 | 131 | 13.1 | | 1201 | 12.7 |
| Attended some college | 117 | 11.7 | 100 | 10.0 | | 913 | 9.7 |
| College degree (4-year) | 398 | 39.8 | 428 | 39.8 | | 4093 | 43.4 |
| Do not know | 255 | 25.5 | 254 | 25.4 | | 2362 | 25.1 |
| Missing | 8 | 0.9 | 9 | 0.9 | | 59 | 0.6 |
| *Free, reduced-price lunch program* | | | | |  |  |  |
| No | 337 | 33.7 | 347 | 34.7 | | 3273 | 34.7 |
| Yes | 488 | 48.8 | 461 | 46.1 | | 4459 | 47.3 |
| Do not know | 172 | 17.2 | 184 | 18.4 | | 1628 | 17.3 |
| Missing | 3 | 0.3 | 8 | 0.8 | | 66 | 0.7 |

**Supplmental Material Table 3: Alpha and Omega Reliability Coefficients for Sample 1**

| Overall | α | ω | Domains | α | ω | Subdomains | α | ω |
| --- | --- | --- | --- | --- | --- | --- | --- | --- |
| SEHS-S-2020 Covitality | .96 | .95 | Belief in Self | .88 | .87 | Self-Efficacy | .83 | .83 |
|  |  |  |  |  |  | Self-Awareness | .79 | .80 |
|  |  |  |  |  |  | Persistence | .76 | .76 |
|  |  |  | Belief in Others | .87 | .85 | School Support | .84 | .84 |
|  |  |  |  |  |  | Family Support | .91 | .91 |
|  |  |  |  |  |  | Peer Support | .91 | .91 |
|  |  |  | Emotional Competence | .87 | .87 | Emotional Regulation | .79 | .79 |
|  |  |  |  |  |  | Empathy | .85 | .86 |
|  |  |  |  |  |  | Self-Control | .70 | .70 |
|  |  |  | Engaged Living | .94 | .93 | Optimism | .87 | .88 |
|  |  |  |  |  |  | Gratitude | .91 | .92 |
|  |  |  |  |  |  | Zest | .95 | .95 |

**Supplemental Material Table 4****: Social Emotional Health Survey-Secondary (SEHS-S-2020) Items**

| **Directions:** You are being asked to take a survey about how you have felt over the past few weeks. Your school is doing this survey to better understand your school experiences. With this information, your school wants to provide support to help improve your school experiences. Read each item and choose the response that best describes you. Please respond honestly. There are no right or wrong answers. You can skip questions you don’t want to answer.  Response options: 0 = Not at all true, 1 = A little true, 2 = Pretty much true, 3 = Very much true |
| --- |

1. I can work out my problems.
2. I can do most things if I try.
3. There are many things that I do well.
4. There is a purpose to my life.
5. I understand why I do what I do.
6. I understand my moods and feelings.
7. When I do not understand something, I ask the teacher again and again until I understand.
8. I try to answer all the questions asked in class.
9. When I try to solve a math problem, I will not stop until I find a final solution.
10. At my school, there is a teacher or some other adult who always wants me to do my best.
11. At my school, there is a teacher or some other adult who listens to me when I have something to say.
12. At my school, there is a teacher or some other adult who believes that I will be a success.
13. My family members really help and support one another.
14. My family really gets along well with each other.
15. There is a feeling of togetherness in my family.
16. I have a friend my age who really cares about me.
17. I have a friend my age who talks with me about my problems.
18. I have a friend my age who helps me when I’m having a hard time.
19. I accept responsibility for my actions.
20. When I make a mistake, I admit it.
21. I can deal with being told no.
22. I feel bad when someone gets their feelings hurt.
23. I try to understand what other people go through.
24. I try to understand how other people feel and think.
25. I can wait for what I want.
26. I don’t bother others when they are busy.
27. I think before I act.
28. On most days I feel, grateful
29. On most days I feel, thankful
30. On most days I feel, appreciative
31. On most days I feel energetic
32. On most days I feel active
33. On most days I feel enthusiastic
34. Each day I look forward to having a lot of fun.
35. Overall, I expect more good things to happen to me than bad things.
36. I usually expect to have a good day.

Belief in Self (Items 1-9): Self-Efficacy (1-3), Self-Awareness (4-6), Persistence (7-9).

Belief in Others (Items 10-18): School Support (10-12), Family Support (13-15), Peer Support (16-18).

Emotional Competence (Items 19-27): Emotion Regulation (19-21), Empathy (22-24), Self-Control (25-27). Engaged Living (Items 28-36): Gratitude (28-30), Zest (31-33), Optimism (34-36).

Additional information:<https://www.covitalityucsb.info/> mfurlong@ucsb.edu
